# Immunotherapeutic effect of BCG-polysaccharide nucleic acid powder on *Mycobacterium tuberculosis*-infected mice using microneedle patches

**DOI:** 10.1080/10717544.2017.1391892

**Published:** 2017-10-25

**Authors:** Qinying Yan, Houming Liu, Zhigang Cheng, Yun Xue, Zhide Cheng, Xuyong Dai, Wanshui Shan, Fan Chen

**Affiliations:** a College of Pharmaceutical Sciences, Zhejiang University of Technology, Hangzhou, PR China;; b Laboratory of Shenzhen Third People’s Hospital, Shenzhen, PR China;; c Wuhan Biocause Pharmaceutical Development Co. Ltd, Wuhan, PR China;; d Lab of Medical Engineering, College of Medical Technology and Engineering, Henan University of Science and Technology, Luoyang, PR China;; e Hubei Collaborative Innovation Center for Green Transformation of Bio-Resources, Life Sciences School of Hubei University, Wuhan, PR China

**Keywords:** Immunotherapy, BCG-PSN, tuberculosis, powder, microneedle patch

## Abstract

Polysaccharide nucleic acid fractions of bacillus Calmette–Guérin, termed BCG-PSN, have traditionally been used as immunomodulators in the treatment of dermatitis and allergic diseases. While the sales of injectable BCG-PSN have shown steady growth in recent years, no reports of using BCG-PSN powder or its immunotherapeutic effects exist. Here, BCG-PSN powder was applied directly to the skin to evaluate the immunotherapeutic effects on mice infected with *Mycobacterium tuberculosis* (MTB). In total, 34 μg of BCG-PSN powder could be loaded into a microneedle patch (MNP). Mice receiving BCG-PSN powder delivered via MNP exhibited significantly increased IFN-γ and TNF-α production in peripheral blood CD4 + T cells and improved pathological changes in their lungs and spleens compared to control group mice. The immunotherapeutic effect of BCG-PSN powder delivered via MNP was better than that delivered via intramuscular injection to some extent. Furthermore, MNPs eliminate the side effects of syringes, and this study demonstrated that BCG-PSN can be clinically administrated in powder form.

## Introduction

Tuberculosis (TB) remains a leading cause of mortality among bacterial diseases, as 10.4 million new TB cases, with 1.4 million being killed, were estimated in 2015 alone (World Health Organization [WHO], 2016). The numbers of first- and second-line anti-TB drugs have increased greatly in recent years to combat the emergence of resistant strains (Munsiff et al., 2003; Matteelli et al., 2014). Moreover, totally drug-resistant tuberculosis (TDR-TB) strains were reported in Italy in 2006 and later reported in Iran, India and South Africa (Parida et al., 2015). While there is an urgent need for efficacious drugs against TB (Günther, 2014), current methods for managing TB infection require treatment for at least 6 months, which leads to noncompliance-related issues and results in the emergence of drug-resistant TB strains. Immunotherapy may be an alternative treatment method prior to new, effective drugs being available for general use. Immunotherapies are thought to modulate the immune systems of patients with latent TB infection or active disease, enabling better control of *Mycobacterium tuberculosis* (MTB) replication. In this study, bacillus Calmette–Guérin polysaccharide nucleic acid (BCG-PSN) powder was investigated as a treatment option for mice infected with MTB.

BCG-PSN is an immunomodulator approved by the State Food and Drug Administration (SFDA) of China (Liu et al., 2016) that is extracted from BCG using the hot phenol method and purified before being dissolved in 0.9% NaCl. BCG-PSN has proven to be effective for the treatment of atopic dermatitis and other allergic diseases (Wang et al., 2014) and is applied in the clinic by intramuscular injection, which usually requires several months. Considering the abundant antigen-presenting cells in skin layers (Kupper & Fuhlbrigge, 2004), better immunotherapeutic effects may be achieved by delivering BCG-PSN to the skin. In the past decade, various microneedles, including single microneedles and microneedle arrays (MNAs), have been developed for skin vaccination or drug delivery in a minimally invasive and painless manner (Gill et al., 2008; Sullivan et al., 2008; Donnelly et al., 2010; Kim et al., 2012). Although no studies on delivering anti-TB drugs via microneedle have been reported, BCG vaccines coated onto metal MNAs have been investigated (Hiraishi et al., 2011). Most vaccines and drugs are freeze-dried and thus require reconstitution before injection, which often incurs errors due to inappropriate opening of the glass ampoule to access the powder or mishandling the dilution. The delivery of powdered agents to the skin for vaccination and allergen immunotherapy has been reported (Chen et al., 2014; Kumar et al., 2016). In addition to being lesion-free, BCG powder vaccines delivered via microneedles have other advantages, such as long storage life and high reactogenicity (Chen et al., 2017).

In this study, we hypothesized that delivering BCG-PSN powder directly to the skin using microneedles would have an improved immunotherapeutic effect compared to that of intramuscular (IM) injection. We tested this hypothesis by investigating the immunotherapeutic efficacy of BCG-PSN powder delivered via microneedle, revealing that BCG-PSN powder delivered in this manner could inhibit bacterial growth, increase the secretion of IFN-γ and TNF-α in CD4 + T cells, and protect against infection more efficiently than BCG-PSN delivered via conventional IM injection.

## Materials and methods

### Mycobacteria and animals

The MTB strain H37Rv [ATCC 93009] was purchased from the Beijing Biological Product Institute, and BCG (D2PB302) (Beijing, China) was preserved in the Biosafety Laboratory of Zhejiang University of Technology. Bacteria were cultured and maintained on Lowenstein–Jensen (L–J) medium and harvested while in log phase growth. Bacilli were washed in 0.05% Tween-80 saline and triturated to uniformity before use. Five-week-old female BALB/c mice missing specific pathogens were obtained from Zhejiang Academy of Medical Science (Hangzhou, China). Mice were maintained under infection barrier conditions in a negative pressure animal room and fed a sterile commercial mouse diet. All studies were reviewed and approved by the Institutional Animal Care and Use Committee of Zhejiang University of Technology.

### Fabrication of BCG-PSN powder-laden microneedles

Microneedle patches (MNPs) were fabricated as previously described (Chen et al., 2017). In brief, a female polydimethylsiloxane (PDMS, Dow Corning, and Sylgard 184) mold with a 6 × 9 MNA was prepared using each microneedle at a height of 200 μm height and a base diameter of 100 μm. A 15% sodium hyaluronate (HA, Lifecore, Chaska, MN, 024477) solution in distilled water was added to the female PDMS mold and centrifuged to fabricate microneedles containing cavities. Then, the microneedles were filled with BCG-PSN powder (a gift from Zhejiang Wansheng Pharmaceutical Co., Ltd., Zhejiang, China) and added to 10% HA to form the patch. To visualize the powder packed in the microneedles, sulforhodamine B (SRB) powder was added to the microneedles cavities, and HA solution mixed with a small amount of fluorescein isothiocyanate (FITC, Sigma-Aldrich, St. Louis, MO) was used to fabricate the hollow MNP as described above. The resulting microneedles were scanned by two photon confocal microscopy, showing that the SRB powder was loaded into the microneedle cavities.

### Quantification of BCG-PSN in the microneedles

To quantify the amount of BCG-PSN powder encapsulated in each microneedle in the array, a standard curve was established first by measuring the absorbance of nucleic acids in serially diluted BCG-PSN using a Nanodrop 8000 spectrophotometer (Thermo Scientific, Waltham, MA). Next, nine microneedles were randomly cut from each patch and diluted to measure the nucleic acid absorbance. In addition, the loading capacity of each microneedle was calculated according to the mean value and the standard curve. The experiment was repeated three times using six microneedle patches in each test.

### Infected mouse model and immunotherapy

Five-week-old female BALB/c mice missing specific pathogens were purchased from the Shenzhen Center for Disease Control and Prevention. In total, 30 female BALB/c mice were challenged with 5 × 10^5^ H37Rv colony forming units (CFUs) via tail vein injection and then randomly divided into three groups: the IM injection group, the MNP delivery group, and the control group. One week after infection, mice from the three groups were injected with either BCG-PSN (34 μg/100 μl) or saline (100 μl) via their tibialis anterior muscle or treated with BCG-PSN powder delivered transcutaneously via the microneedle patch applied to low dorsal skin. Each mouse received one single treatment per day, and the treatment continued for one week.

### Intracellular cytokine production in peripheral blood T lymphocytes

Four weeks after infection, blood samples were collected from the orbital sinus cavities of the mice to evaluate the immune responses elicited by BCG-PSN. After the lysis of red blood cells using ammonium-chloride-potassium (ACK) buffer, peripheral cells were isolated via centrifugation and subsequently cultured in Dulbecco’s Modified Eagle’s Medium (DMEM, Sigma) supplemented with 10% fetal calf serum (FCS), 100 U/ml penicillin, and 100 mg/ml streptomycin (Gibco, Carlsbad, CA). Single-cell suspensions were stimulated for 16 h at 37 °C and 5% CO_2_ using BCG bacillus outer membrane extract (Rodriguez et al., 2011; Tirado et al., 2015) supplied via the DMEM as described above at a final concentration of 5 μg/ml to a cell density of 2 × 10^6^ cells/ml. The cells were continuously cultured for another 5 h in the presence of GolgiPlug (BD Biosciences, Franklin Lakes, NJ) per the manufacturer’s instructions. The activated cells were surface-stained with a FITC-labeled anti-CD4 antibody, permeabilized with Cytofix/Cytoperm buffer (BD Biosciences), and intracellularly stained with PE-anti-IFN-γ and APC-anti-TNF-α antibodies (BD Biosciences). The numbers of specific cell types were quantified using a FACSAria flow cytometer (Beckman, Brea, CA).

### Bacteria counts and histopathological observations

Mice were sacrificed after the blood samples were collected, and their lungs, and spleens were dissected and ground in 1 ml of PBS. The lung and spleen mouse tissue suspensions were serially diluted 10-fold and cultured on L–J medium plates at 37 °C for 4 weeks. MTB colonies on the medium were counted, and the results are shown as the log CFU per organ. Portions of lungs from three mice of each group were soaked in 4% paraformaldehyde for more than 24 h, and pathological sections were examined via hematoxylin and eosin (HE) and acid-fast staining.

### Statistical analysis

The data were analyzed by one-way analysis of variance (ANOVA) for comparisons among multiple groups and by Student’s t-tests for comparisons between two groups with GraphPad Prism version 6 software (GraphPad Software, La Jolla, CA). Mycobacterial counts were log_10_-transformed and analyzed using one-way ANOVA followed by Bonferroni’s test. *p* Values < .05 were considered significant.

## Results

### Microneedle patch and powder-laden microneedles

**Figure 1.**
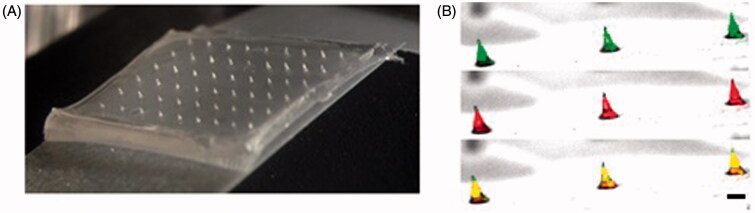
Characterization of powder-laden MNPs. (A) Microneedle patch with a 6 × 9 array. (B) Microneedles labeled with FITC and loaded with SRB powder were observed under a confocal microscope (10×). Scale bar, 100 μm.

Microneedle patches were made with sodium HA, a common ingredient in skin or skin care products that has no adverse reactions (Fraser et al., 1997). The microneedle patches were composed of 54 microneedles in a 6 × 9 pattern and adhered to tape (Figure 1(A)). To verify that BCG-PSN powder was effectively loaded in the microneedle cavities, SRB powder was loaded as a reference substance, and an HA solution mixed with FITC was added to a FITC-labeled microneedle. Confocal microscopy clearly demonstrated that SRB was successfully loaded into the microneedle cavities (Figure 1(B)).

### Quantitative analysis of BCG-PSN powder

**Figure 2.**
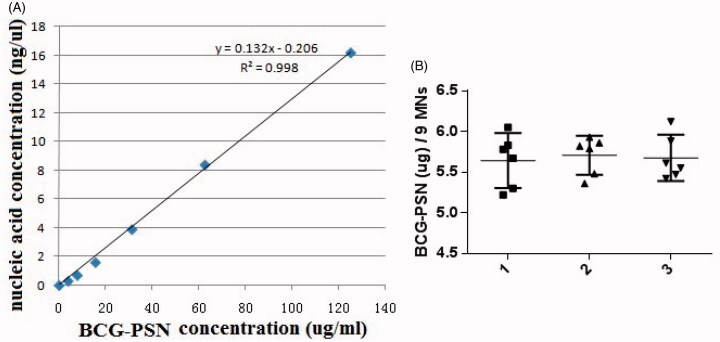
Determination of drug loading capacity. (A) Standard curve of absorbances at 260 nm vs. varying amounts of BCG-PSN powder dilutions. (B) The amount of BCG-PSN powder within an MNP was evaluated based on the standard curve shown in A. Each symbol represents the amount of BCG-PSN in nine microneedles cut from each patch, and the horizontal lines indicate the means of 6 patches with a 95% CI (confidence interval). The results of three independent experiments, designated 1, 2, and 3, are shown. In total, six MNPs and nine microneedles were used in each experiment.

The amounts of BCG-PSN powder encapsulated in the MNPs were estimated using a standard nucleic acid absorbance curve with serially diluted BCG-PSN solutions (Figure 2(A)). Nine BCG-PSN-packed microneedles from each patch were randomly cut and dissolved in dH_2_0. The average amount of BCG-PSN powder per microneedle from a total of nine microneedles in six different patches was approximately 0.63 µg with a 95% CI (Figure 2(B)). Similar results were obtained from three independent experiments, with six microneedle patches being analyzed in each experiment. These results indicated that approximately 34 µg of BCG-PSN powder could be delivered to the skin using a single patch with a 6 × 9 pattern.

### Cytokine production in mouse spleens and lungs

**Figure 3.**
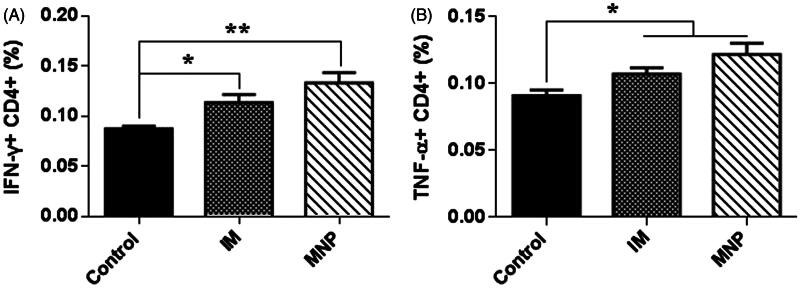
IFN-γ and TNF-α production in peripheral blood CD4 + T cells following BCG-PSN treatment. The data are representative of two independent studies with similar results and expressed as the mean ± SEM (*n* = 6 for each experiment). *p < .05 and **p < .01.

To understand the immunomodulatory effects of BCG-PSN, the cytokines IFN-γ and TNF-α were measured in peripheral blood T lymphocytes 2 weeks after administration of the immunotherapy. After *ex vivo* stimulation with BCG bacillus outer membrane extract for 21 h, CD4 + T cells in peripheral blood produced significantly higher levels of IFN-γ and TNF-α in the presence of BCG-PSN than in the absence of BCG-PSN (Figure 3(A,B)). When compared to the control group, the MNP delivery group secreted more IFN-γ than the IM injection group (*p* < .01 vs. *p* < .05). These results suggested that BCG-PSN could effectively adjust T cell immune functions, and BCG-PSN delivered via the MNP showed better immunomodulatory effects than that delivered via IM injection.

### Immunotherapeutic effect of BCG-PSN

**Figure 4.**
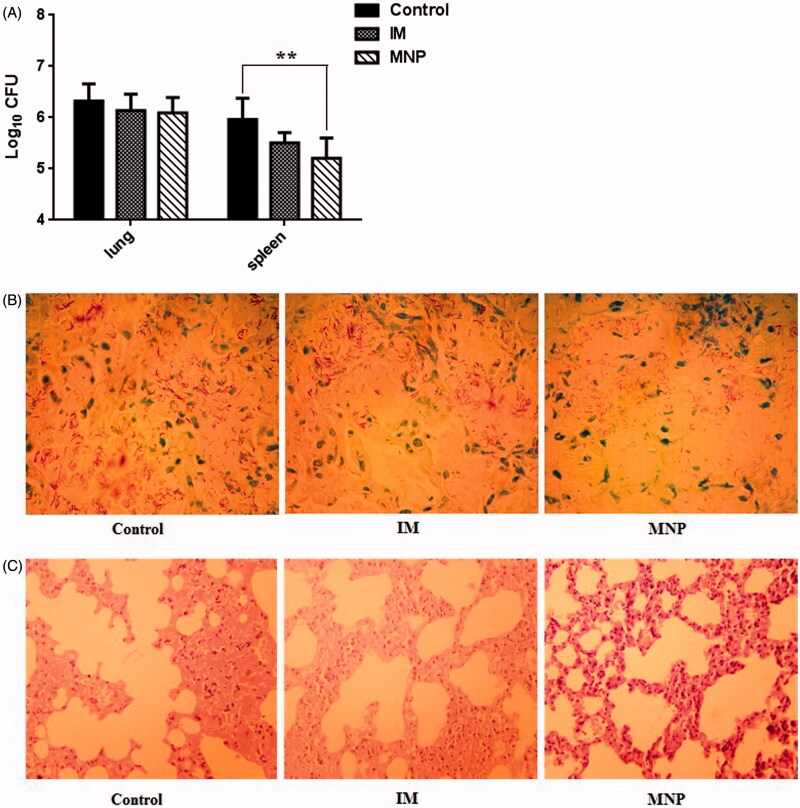
Evaluation of immunotherapeutic effects *in vivo*. (A) Determination of CFUs from the lungs and spleens of infected mice from each group. Colony counts were determined after four-week incubation at 37 °C and are represented as the log CFU value. Data are presented as the mean ± SEM (*n* = 6 for each experiment). **p < .01. (B) Acid-fast staining of mouse lung biopsy specimens (1000×). (C) Histopathological examination of mouse lung biopsy specimens (HE, 40×).

The bacterial numbers in lungs and spleens were decreased in both the IM injection group and the MNP delivery group compared to those in the control group. However, there were no significant differences between the IM and control groups in the lungs or the spleen. However, the number of bacteria in the spleen was significantly decreased in the MNP group compared to that in the control group (Figure 4(A)). Moreover, compared with the control group, the density of acid-fast bacillus in the lungs was reduced in the MNP delivery group (Figure 4(B); the acid-fast stained slice of only one mouse from each group is shown, while the other five slices from each group are shown in the supplementary data). Histopathological examination of lungs in the control group mice showed a marked inflammatory reaction, including alveoli decreased and fusion. However, when treated with BCG-PSN, both the IM injection group and the MNP delivery group showed less pulmonary alveoli fusion and swelling and more prominent air spaces (Figure 4(C)) (The lung slice of only one mouse from each group is shown, while the other five slices from each group are shown in the supplementary data). These results demonstrated that BCG-PSN has an immunotherapeutic effect against TB, and the effect can be attained by the transdermal delivery route.

## Discussion

BCG-PSN macromolecules have been used for the prevention and treatment of chronic bronchitis, colds, and asthma for years in China, and they may possess immunoregulatory active materials, including polysaccharides, nucleic acids, proteins, and lipids. These biomacromolecules participate in multiple biological activities, such as cell-to-cell communication and immune regulation (Wei et al., 2015). As the number of BCG-PSN applications increases, BCG-PSN can reportedly treat erosive oral lichen planus, act as an adjuvant for HIV vaccines, and even inhibit the growth of nasopharyngeal carcinoma cells (Xiong et al., 2009; Sun et al., 2013; Xu et al., 2015).

However, disadvantages of BCG-PSN injections include long treatment cycles and some side effects, such as erythema, nodules, and slight fever. Therefore, BCG-PSN injection using a hypodermic needle or syringe is a deterrent for patient acceptance due to needle phobia and pain associated with the injection (Mitragotri, 2005). In this study, BCG-PSN powder was loaded onto dissolving microneedles (Figure 1(A,B)) and delivered to the skin directly, thus avoiding side effects caused by needles and syringes. In total, 34 µg of BCG-PSN powder (Figure 2(A,B)) could be delivered to the skin using a single patch, which was unparalleled to any other dissolvable or coated microneedle arrays of similar microneedle size and density. After one week of treatment with BCG-PSN powder (Figure 3(A,B)), peripheral blood CD4 + T cells produced more IFN-γ and TNF-α, which are important for controlling MTB infections (Flynn et al., 1993; Saunders et al., 2004). Interestingly, when both were compared to the control group, the MNP delivery group secreted more IFN-γ than the IM injection group (Figure 3(A)). Thus, BCG-PSN delivered intradermally via an MNP may elicit a better immunomodulatory response than that delivered intramuscularly. Moreover, the bacterial population in the spleen was decreased in the presence of BCG-PSN (Figure 4(A)). Acid-fast stained lung slices showed decreased bacterial numbers to some extent in both the IM and MNP groups (Figure 4(B) and supplementary data). In addition, the immunotherapeutic effect of BCG-PSN was also observed in pathological tissue slices (Figure 4(C) and supplementary data). These data demonstrated that BCG-PSN powder can be directly applied via MNP, and its immunotherapeutic effect when delivered via MNP is similar or better than that achieved with IM injection.

Currently, many drugs are packaged as freeze-dried powders with diluents in two different ampoules and must then be reconstituted before injection. The reconstitution procedure is time-consuming, and using the wrong diluent may cause serious problems. In addition, traditional injection methods require the disposal of sharp biohazards needles and increase the burden of transportation. These limitations can be overcome by using drugs in powder form directly. Here, MNPs could sufficiently deliver BCG-PSN powder to the skin, which was advantageously lesion-free and painless. This method of drug administration serves as an alternative to traditional methods during clinical therapy, especially for patients who require long-term drug treatment.

## Conclusions

Many drugs or vaccines are currently packaged as freeze-dried powders. However, the reconstitution procedure is time-consuming, and using the wrong diluent may cause serious problems. This study demonstrated that BCG-PSN could be clinically administered in powder form using a needle-free and painless microneedle patch, and the immunotherapeutic effect of this method was similar or better than that achieved using IM injection.

## Supplementary Material

IDRD_Chen_et_al_Supplemental_Content.zip
