# Supplementary figures and images for: Immunotherapeutic effect of BCG-polysaccharide nucleic acid powder on Mycobacterium tuberculosis-infected mice using microneedle patches
Source: Drug Deliv. 2017 Oct 25;24(1):1648–53. doi: 10.1080/10717544.2017.1391892 (PMC8241181; doi:10.1080/10717544.2017.1391892)

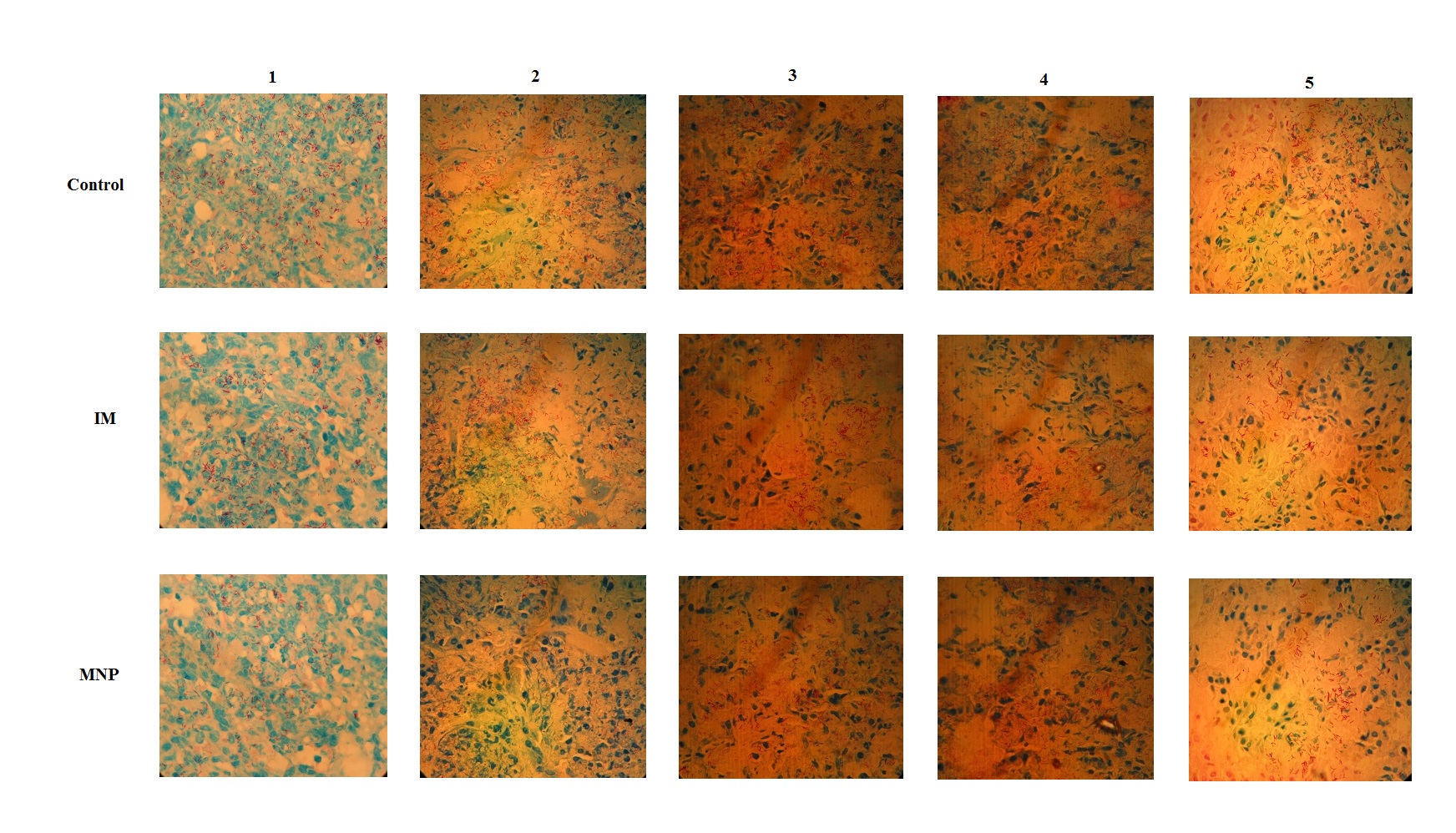

Supplement: IDRD_Chen_et_al_Supplemental_Content.zip [file IDRD_A_1391892_SM5830.zip › Sup-figure 1.jpg]

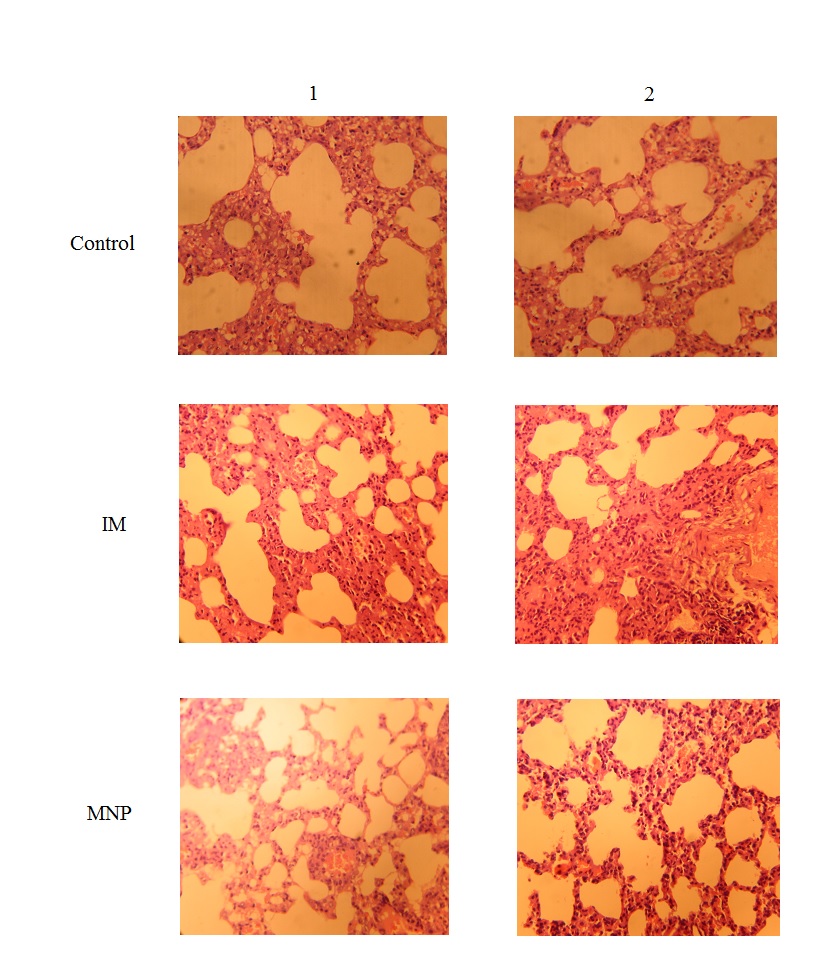

Supplement: IDRD_Chen_et_al_Supplemental_Content.zip [file IDRD_A_1391892_SM5830.zip › Sup-figure 2.jpg]
